# Curling Up With a Good E-Book: Mother-Child Shared Story Reading on Screen or Paper Affects Embodied Interaction and Warmth

**DOI:** 10.3389/fpsyg.2016.01951

**Published:** 2016-12-15

**Authors:** Nicola Yuill, Alex F. Martin

**Affiliations:** Children and Technology Laboratory, School of Psychology, University of SussexFalmer, UK

**Keywords:** shared-reading, tablets, embodied cognition, synchrony, affect, human–computer interaction (HCI)

## Abstract

This study compared changes in cognitive, affective, and postural aspects of interaction during shared mother and child book reading on screen and on paper. Readers commonly express strong preferences for reading on paper, but several studies have shown marginal, if any, effects of text medium on cognitive outcomes such as recall. Shared reading with a parent is an engaging, affective and embodied experience across time, as well as a cognitive task, so it is important to understand how paper vs. screen affects broader aspects of these shared experiences. Mid-childhood sees a steep rise in screen use alongside a shift from shared to independent reading. We assessed how the medium of paper or screen might alter children’s shared reading experiences at this transitional age. Twenty-four 7- to 9-year-old children and their mothers were videotaped sharing a story book for 8 min in each of four conditions: mother or child as reader, paper, or tablet screen as medium. We rated videotapes for interaction warmth and child engagement by minute and analyzed dyadic postural synchrony, mothers’ commentaries and quality of children’s recall, also interviewing participants about their experiences of reading and technology. We found no differences in recall quality but interaction warmth was lower for screen than for paper, and dropped over time, notably when children read on screen. Interactions also differed between mother-led and child-led reading. We propose that mother - child posture for paper reading supported more shared activity and argue that cultural affordances of screens, together with physical differences between devices, support different behaviors that affect shared engagement, with implications for the design and use of digital technology at home and at school. We advocate studying embodied and affective aspects of shared reading to understand the overall implications of screens in children’s transition to independent reading.

## Introduction

When children share reading with their parents, on the road to becoming independent readers, does it matter whether they share stories using a traditional paper book or a tablet screen? Intense media interest surrounds the question of whether reading on screen differs from reading on paper, and there is now a small but growing literature on the topic. Many adults express a preference for paper (Pew Research Center, 2016) and sales of paper books have recently shown a small rise as e-book sales have slightly declined (Publishers Association, 2015). Where children are concerned, there has been strong concern about the amount of ‘screen time’, with fears that children reading from screens may not derive the same benefits as those reading from paper, and that digital devices will discourage children from reading for pleasure. In one recent report, 74% of parents said they would rather their child read a print book than an e-book, and 50% of parents of 5- to 7-year-olds worry about their child’s excessive use of screens (Egmont, 2013). Conversely, a report from the National Literary Trust suggested that using e-books may increase the motivation and reading skills of young readers, particularly poorer performing boys (Picton and Clark, 2015). These questions have practical importance because parental involvement in reading influences children’s later language and literacy development (e.g., Bus et al., 1995; Senechal and LeFevre, 2002) and is entwined with the attachment relationship of parent and child (Bus and van Ijzendoorn, 1995). Shared reading is a potent environment for the sort of positive parent-child interaction that can contribute to socio-emotional development, as well as literacy (Aram and Aviram, 2009).

As technology becomes increasingly pervasive in children’s lives, the question of how digital technology affects their literacy and enjoyment of stories becomes more pressing. Figures from the UK communications regulator Ofcom (2015) show that the use of digital technology for entertainment is now something that even the youngest toddler encounters. The use of technology is also widespread in schools, so children’s experience of reading, both at home and at school, is increasingly through the medium of a screen.

Research on children’s reading from screens has focused, understandably, on young children who are just learning to decode text, and most of these studies therefore involve adult-led shared reading. Children’s early experiences of books, whether on paper or on screen, are thus typically triadic interactions – reading device, child and adult. An increasingly influential approach in the field of human-computer interaction (HCI) is that of ‘embodied interaction’, ‘the creation, manipulation, and sharing of meaning through engaged interaction with artifacts’ (Dourish, 2004, p. 126), emphasizing the everyday social practices and physical reality through which people interact with technology, involving shared awareness, construction of meaning and emotions. Joint book-reading fundamentally involves the shared construction of meaning between adult and child, with the different opportunities and constraints provided by books or screens, both in terms of their physical properties and their social significance. Studying shared reading with this perspective in mind can help us see the contribution of different aspects of the natural situations in which joint reading happens. Potential differences in cognitive outcomes, such as comprehension, are without doubt important, but research also needs to investigate affective, interactional, and embodied factors that are central to the experience of early shared reading: child engagement in the story, interaction warmth and postural synchrony, as evidenced by how the two readers physically position themselves in relation to each other and the device. This wider compass is important because typical early reading involves triadic interaction in existing close relationships, in a cultural context, rather than being ahistorical, individual encounters between brains and words.

For traditional paper books, the typical transition period from shared reading to reading independently and alone usually occurs around the age of 7 to 9. At this point, children become able to choose books for themselves, to develop preferences, to start reading ‘chapter books’ independently, and to decide how much time they wish to spend reading. This age also marks a gradual shift from parent-led to child-led reading, with parents in a Book Trust survey reporting a drop in reading bedtime stories to their children, from 86% at 5-years-old, to just 38% at 11-years-old (Book Trust, 2016). Taking a school book home for shared reading is standard practice in the UK and elsewhere, supporting both literacy development and shared enjoyment through parent-child interaction. The significance of this phase in literacy development is demonstrated through statistics on book and computer use; book reading drops sharply at about the same rate as digital media consumption rises: (Egmont, 2013). This period of concurrent transitions, from shared to independent reading, and from reading for pleasure to multi-media usage, makes this age group of particular interest in investigating differences in reading experience between paper and screen.

In this study, we addressed potential differences between shared reading of digital and paper texts, when children are reading and being read to, in four inter-related areas:

*Cognitive*: Do children differ in the quality of their descriptive and structural recall for texts read on screen and on paper? Do they differ in their attentional engagement with the story between the two media, when reading and when being read to?

*Interactive and affective*: Are there differences in the warmth of mother-child interactions between screen and paper media, and depending on who is reading? Do mothers provide different kinds of verbal support according to medium and reader?

*Postural synchrony*: Are there coherent differences in the physical positioning of mother and child when reading from screens vs. paper?

*Attitudinal*: do mothers and children have different experiences with, and attitudes to shared and independent reading on screen and on paper?

The literature on reading traditional paper books with children ranging from toddlerhood to around age 10 focuses mainly on cognitive factors, and shows that shared reading aids children’s learning, e.g., of vocabulary (for a meta-analysis see Flack, Field, and Horst, 2016, under review). Dialogic reading styles, where the adult engages in conversation about the story, are particularly helpful for learning and engagement (e.g., Reese and Cox, 1999), and for engendering a love of reading (Bus, 2001). Most comparative studies with e-books involve digital devices designed to support independent reading through audio, multimedia content, and games (e.g., see Bus et al., 2015). These enhanced e-books are generally not designed for shared reading, and can hence become frustrating for adult reading partners (see Chiong et al., 2012). Although there is less evidence comparing children’s reading from paper to more basic e-books (text on screen with minimal extra features), there is some agreement within the existing studies: Chiong et al. (2012) found lower story comprehension in 3- to 5-year-olds reading a science-themed book with a parent from an iPad than from a paper book, and Krcmar and Cingel (2014) found a small but significant drop in comprehension for pre-schoolers reading with a parent on an iPad compared with paper. In both cases, the screen reading prompted more conversation about the processes of reading, likely at the expense of story-relevant comments. Similar findings about conversation type and comprehension were reported for 3- to 5-year-olds co-reading with adults (Parish-Morris et al., 2013), and also in a comparison of parents reading stories on paper vs. laptop with 4-year-olds (Lauricella et al., 2014), although this last study found no significant difference in comprehension. The ‘traditional’ books in these studies were generally unornamented paper books, although books aimed at younger children in particular often have features such as texture, sound, pop-ups or flaps: books with flaps were compared to e-books by Moody et al. (2010) although no comment was made specifically on the role of these interactive paper features.

There is an abiding feeling expressed by many adults that reading an e-book provides a different ‘feel’ and sense of engagement from reading on paper. There are some cognitively helpful affordances of paper, such as for note-taking and studying, that are not well-replicated in electronic media (O’Hara and Sellen, 1997) but could there also be differences in the child’s engagement with narrative during shared reading on paper vs. on screen? As noted above, some studies have included measures of engagement. Lauricella et al. (2014) compared parent-led shared reading of a print book and a laptop e-book in parents of 4-year-olds, and coded parent-child engagement (a broad measure combining video ratings of active vs. passive parent involvement, mutuality of communication, parental success in engaging their child, and degree of conversational turn-taking). They found higher engagement for the e-book than for the paper book, similar to findings by Chiong et al. (2012) on children’s engagement with paper vs. tablet books in 3- to 6-year-olds. However, engagement as measured in these studies included physical interaction, such as page-turning processes, using a mouse or touching hotspots, which would likely be required more for touchscreens or computer mice than for paper, and it is possible that heightened excitement because of novel technology use might also be seen as greater engagement. Measures of engagement that focus more on attentional engagement with the story than on physical interaction with a device may not find the same advantages for e-books. In fact, Chiong et al. (2012) measured ‘overall engagement’ including parent-child interaction and enjoyment, and found more such engagement for a print book than an e-book. It is therefore unclear whether a child’s engagement with a story differs between parent-led shared reading on paper and on screen. We address this issue in our study by using a measure of child engagement based not on physical movement prompted by the device, but on the child’s attentional engagement with the story.

The link between affect and shared reading has been recognized in research into early (pre-school) literacy, primarily in relation to mother-child attachment security (Bus and Van Ijzendoorn, 1988; Frosch et al., 2001; Daly et al., 2015). Despite this recognition, assessment of affective aspects of shared reading in print and on screen seems to have been neglected, particularly in studies beyond infancy. Techniques to measure characteristics such as warmth are easily available in the well-established literature on family interactions involving young children, so we adapted a measure of warmth (positive affect) from the widely used Parent-Child Interaction System, PARCHISY (Deater-Deckard et al., 1997). Given the lack of previous research that focuses on warmth independently of other aspects of general interaction, we did not make predictions about differences in interactional warmth by medium, or by reader. We note in respect of reader, though, that the adult tends to have a different role when reading or listening to a child read. While the adult is in both situations in a didactic, expert role, we would expect the focus in child-led reading to be more on supporting the child’s decoding than on story discussion, and this might make for lower warmth.

Warmth and attunement to the needs of the child are underlying features of the dialogic reading style, so analyzing the ways that mothers talk with their children during shared reading should illuminate ways that the type of medium and reader influence shared reading. Several previous studies of e-books have analyzed the nature of adults’ comments during shared reading. Perhaps unsurprisingly, adults make more comments about the mechanics of reading (e.g., about page turns or touching screens), and fewer comments about the story itself, such as vocabulary, for e-books than paper (Chiong et al., 2012; Krcmar and Cingel, 2014; Lauricella et al., 2014). Because we looked at both mother and child as reader, we separated out mothers’ comments about specific vocabulary and about the story more generally. We expected that mothers would give more support for vocabulary when the child was reading, and this might be at the expense of broader story-related comments.

Physical positioning and interactional synchrony are intrinsic, but largely ignored, aspects of shared reading. We know that more broadly, dyadic synchrony is fundamentally involved in cognitive, social and emotional development (Harrist and Waugh, 2002) and exerts a fundamental influence on the tenor and warmth of interactions. We could find little or no evidence on the role of posture and synchrony in shared reading, but we predicted that postural synchrony would have an important role to play in shared reading interactions, with the potential to illuminate differences in the experiences of reading on screen and paper. Lauricella et al. (2014) described parent-child interaction for paper and screen in terms of how parents arranged the seating. However, the use of a laptop for the e-book affected positioning in a specific way, because the laptop generally had to be placed on a table, and since there was one mouse, control could not be shared. They found that half the children controlled the mouse in the e-book condition, increasing those children’s physical engagement. In the present study we aimed to reduce variation introduced by device demands by using a tablet and book with similar dimensions, which could be held and controlled in similar ways, allowing the assessment of differences in dyadic posture and synchronization between the parent and child. We also compared child-led and parent-led shared reading: given that tablet use tends to be primarily individual, we expected that shared reading with a tablet might pose challenges in sharing the device.

In summary, we compared shared reading of illustrated chapter books between mothers and their 7- to 9-year-old children, on paper or on screen, with the child or the mother as reader, to investigate four aspects of the interactions: cognitive (recall and engagement), interactive warmth and dialog, postural synchrony, and attitudes to and experience with technology.

## Materials and Methods

### Participants

Participants were recruited from 10 classes in four primary schools in a semi-rural region of south-east England, where flyers were put in the book-bags of all 7- to 9-year-old children, inviting them to take part. Twenty-eight families responded to the advert, of whom 26 agreed to take part. Two children were excluded from the final sample; one with dyslexia and one who did not meet the age criterion. Parents gave written, informed consent, children gave assent, and the study was approved by the University ethics committee. In addition, parents gave written consent to use images in training or publications. When the images herein were selected, as a courtesy we obtained additional written consent from the parents.

The final sample consisted of 24 mother-child dyads, all White-British, reflecting the local population. There were 15 boys and 9 girls, with ages ranging from 7.04 to 9.89 years (*M* = 8.60, *SD* = 0.91). All of the mothers were the biological parent. Mothers’ ages ranged from 30.13 to 51.53 years (*M* = 41.66, *SD* = 4.61).

To assess representativeness, parental education and household income were compared with UK Census data (2011): 83.3% of the mothers held a degree level qualification or higher, which is greater than the national average (33.9% of women). Household incomes in the sample covered the whole range, from $0-14,999 to more than $100,000. The median was $55-74,999, greater than the national average ($46,500).

### Design

We used a repeated-measures design, with each pair reading one book progressively through each of the four conditions (Mother-Paper, Child-Paper, Mother-Digital, Child-Digital) for 8 min in each condition. The order of conditions was counter-balanced in such a way that the medium of reading was blocked together, as follows: two paper conditions (mother reads then child reads, or vice versa) followed by two digital conditions (in the same order of who reads), or the digital conditions followed by paper conditions. This design allowed the comparison of overall recall between reading media without the disruption of repeated changes of device.

### Materials

We gave children a choice of two books, both humorous fiction works recommended for children of 7 and over: ‘*You’re a Bad Man, Mr Gum*’ (Stanton, Jelly Pie, London, 177 PP) and ‘*Barry Loser: I am Not a Loser*’ (Smith, Jelly Pie, London, 239 pages). The first chapter of ‘Mr Gum’ had a Flesch reading ease score of 79.3 and the first chapter of ‘Barry Loser’ scored 85.8, on a 1-100 scale with 100 as easiest. The book was presented as a paperback book, measuring 260 mm × 190 mm when open, and on a Microsoft Surface RT, with a reading area of 235 mm × 132 mm. The Surface RT compared with other tablets has low reflectance and a wide viewing angle (DisplayMate Technologies, n.d.). These features are helpful in supporting shared visual access in dyadic reading. The tablet presentation used the Book Bazaar e-reading application, using the ‘Publisher’s Settings’ option which presented the text in Tahoma typeface, providing a visual appearance very similar to the paper format. Both formats provided text and illustrations in black and white on most pages, although the ways illustrations were positioned in text varied because of different reading area sizes and automated formatting by the e-reader. The two formats differed in weight, with the book at 196 g and the Surface, including case, notably heavier at 1,020 g. The participants had all used tablets, though to different extents (see Results), but were equally unfamiliar with the Surface RT.

### Measures

#### Cognitive

##### Reading accuracy

Children’s reading errors were coded for the first 100 words of the child-reading condition in the digital and paper conditions. Because order of presentation varied, the words read were not identical across children, but the books did not differ systematically in readability through the book, so the accuracy over 100 words would not be expected to differ systematically across books. A reading error was defined as a: failed or non-attempt to read a word; mispronunciation; missing or inserted word; or hesitation followed by mother’s intervention.

##### Recall

The experimenter, who was absent during the reading, checked recall at the end of reading in each medium, i.e., after both partners had read on screen, or on paper. The child was asked, ‘Since you’ve been reading the paper/digital book, can you tell me what’s happened in the story?’. This meant that each reader provided recall data twice, once after the two digital conditions and once after the two paper conditions. In order to discourage parental help, the parent was presented with a questionnaire (see below) during the child recall. Any subsequent parent-assisted recall was not included in the child’s recall score.

Given our design, which aimed to support an informal and natural reading experience, children were recalling from different texts and for different amounts of input, depending on the book choice and the natural reading speed of the readers. We therefore did not score recall in terms of amount of information. We instead used 3-point scales to code descriptive detail and narrative coherence independently of the amount recalled, since the latter would depend on reading speed and fluency:

*Richness of descriptive detail*: 1 = no or very little information with little or no descriptive detail (two or fewer descriptive terms); 2 = some information, with three to four descriptive terms; 3 = more than four descriptive terms or details.

*Narrative coherence*: 1 = events or ideas not linked temporally or causally (e.g., listing unconnected ideas) no causal links; 2 = events or ideas linked as lists or with simple temporal terms (e.g., ‘and then’), two or fewer causal links; 3 = More than two causal links between events or ideas.

Two raters blind to condition double-coded 10 (21%) of the recalls, achieving a satisfactory reliability, κ = 0.71, *p* < 0.01.

##### Video coding

Videos were coded by a researcher blind to the aims of the study and double-coded by a second coder for a randomly selected 25% of video sessions. Resulting reliability Kappa statistics are given below.

#### Interactive and Affective

##### Child engagement

Designed to capture child interest in the story, independently of differences that might occur as a consequence of different affordances of the reading device and reader. Engagement was judged from child visual attention, gesture, expression, and verbalization, and coded every minute on a scale from 1 = child distracted from story to 5 = highly engaged with story (κ = 0.93, *p* < 0.001).

##### Interaction warmth

The warmth of the interaction between mother and child was coded every minute on a 5-point scale, adapted from the PARCHISY coding scheme (Deater-Deckard et al., 1997), from 1 = no positive affect expressed to 5 = continuous positive affect (κ = 0.82, *p* < 0.001).

##### Mother comments

All mother verbalizations were coded into one of five categories:

*Mechanical*: referring to the digital or paper book itself, e.g., ‘turn the page’ ‘tap there to turn’.

*Vocabulary*: Giving the meaning of a word, asking the child what a word means, helping the child decode a word, providing the correct pronunciation of a word.

*Story*: Explaining what is happening in the story, asking the child what is happening, extending the story, commenting on the story

*Motivation*: Encouraging child, e.g., ‘well done’ ‘that was tricky!’, keeping the child on task, e.g. ‘concentrate’ ‘pay attention’, ‘you’re here [pointing]’ or re-reading the last sentence the child read, linking to the child’s own experience, e.g., ‘that sounds like your grandad!’.

*Unrelated*: Any utterance unrelated to the story or task.

#### Postural

We inspected screen shots of how participants positioned themselves with the device in each of the four conditions.

#### Attitudinal

##### Interview

Children were asked about their reading preferences and technology use at the end of the reading task. Mothers completed a paper questionnaire on the same topics which also included demographic questions.

##### Procedure

Families were visited at home on a single occasion by the same female researcher and assessed in as naturalistic a way as possible. Visits took place over a 5-week period at the end of the summer term and the first week of the summer holiday. Seven took place during the day, and 17 after school: we inspected the data for differences but did not see a markedly different pattern for children tested in the day. All participants were seated on comfortable sofas in their living room, except for two pairs who sat on chairs at a table. All except five children were seated on the left of the mother: it is likely that this reflected children’s dominant hand preference but we did not check this. The study was explained, and parent consent and child assent gained, before the children were asked to choose which book they wanted to read, and then the pairs were asked to read aloud as they would normally. The tablet was briefly demonstrated just before the relevant reading conditions and there were no serious misunderstandings about its use or operation. The device was offered to the pair, with no instructions as to who should hold it or how they should arrange their positions. Occasionally other family members were present (a sibling as silent onlooker once, a family pet on six occasions) but no other adult humans were present. Participants were randomly allocated to one of the four orders of condition. The four conditions were completed in sequence, with a verbal recall task for the child after the second and fourth conditions. After the reading task the mother completed a paper questionnaire while the child was interviewed orally by the researcher.

Eleven children distributed roughly evenly between conditions and gender wore light activity monitors on a wrist and an ankle, and of these, three also wore a GoPro headcam, as part of a separate study. We could not detect systematic influences of wearing this equipment on behavior, other than occasional reporting of mild discomfort of a headcam and one child remaining relatively still in all conditions when wearing the activity monitors. These variables are not mentioned further.

## Results

### Reading Choice and Accuracy

Book choices were almost evenly divided (13 Barry Loser, 11 Mr. Gum) with no significant gender or age bias in choice. In general, we found no effects of gender, book choice or condition order, and we do not report further on these variables.

Reading errors varied from 5 to 9% of words, indicating that the books were at the appropriate level, and showing no significant differences between book choice or medium of reading, both *F*s(1,16) < 1. Children tended to progress further through Barry Loser (average progress to page 76) than Mr. Gum (average to page 57), as there were fewer words per page on average in Barry Loser.

### Story Recall

There were no significant differences either in richness or coherence of recall according to text medium, each *F*(1,16) < 1, as shown in **Table 1**. There was no significant difference between the two book choices in either score, both *F*s(1,16) < 1. The design did not enable separation of recall data by reader.

**Table 1:** Mean and SD in recall richness and coherence (max = 3) following digital and paper shared reading.

Condition	Recall richness	Recall coherence
Paper	1.33 (0.82)	1.67 (0.82)
Screen	1.50 (0.84)	1.67 (0.52)

### Child Story Engagement

An analysis of variance (ANOVA) for mean scores of child engagement showed a main effect of reading medium, *F*(1,16) = 4.88, *p* < 0.05. There was a small but significant difference, with higher engagement for reading from paper, *M* = 3.50, *SD* = 0.16, than from screen, *M* = 3.31, *SD* = 0.19. There was also a difference by identity of reader, *F*(1,16) = 8.31, *p* < 0.01, with higher engagement when the child was reading, *M* = 3.64, *SD* = 0.19, than when the mother was, *M* = 3.17, *SD* = 0.19. There was no significant interaction of medium and reader, *F*s(1,16) < 1.

### Interaction Warmth

**FIGURE 1:**
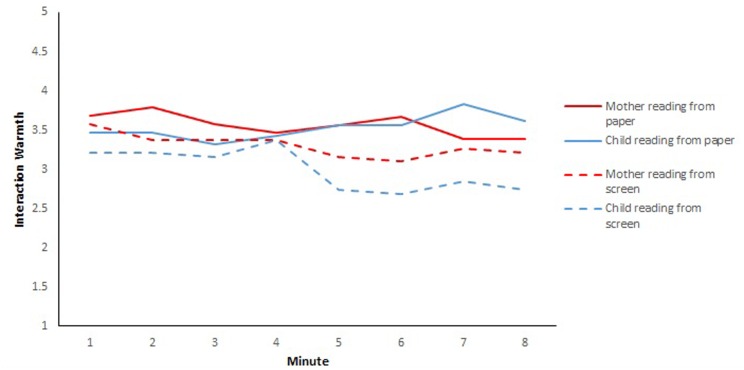
**Interaction warmth per minute for paper vs. screen with mother or child as reader (max = 5)**.

A repeated-measures ANOVA on interaction warmth rating by medium and reader and by minute across the 8 minutes (using Greenhouse-Geisser *F*s to correct for sphericity where required) showed a main effect of reading medium, *F*(1,20) = 5.60, *p* < 0.05, with a slight but significant lower overall warmth for screen reading (*M* = 3.10, *SD* = 0.18) than for paper (*M* = 3.57, *SD* = 0.20). There were no effects for mother vs. child as reader, *F*(1,20) < 1, and a main effect of time, *F*(7,92) = 2.60, *p* < 0.05. These effects were moderated by a significant interaction between medium, reader and time, *F*(7,96.5) = 3.63, *p* < 0.005. The changes in warmth across the sessions are shown in **Figure 1**. There appears to be a marked change for screen reading around halfway through the 8-min session, particularly when children read from screen. We examined the trends over time for the different conditions by running ANOVAs with trend analysis for each condition. There were no significant effects of time on interactive warmth for children reading on paper or for mothers on screen, *F*s < 1. For children reading from screen, there was a main effect of time, *F*(4.4,92.6) = 2.62, *p* < 0.05, with a significant downward linear trend, *F*(1,21) = 6.41, *p* < 0.02, as shown in **Figure 1**. For mothers reading on paper there was also a significant effect of time, *F*(3.78,75.63) = 2.96, *p* < 0.05, with both a linear trend *F*(1,20 = 5.99, *p* < 0.02) and a 5th order trend, *F*(1,20) = 11.13, *p* < 0.005) which we did not seek to interpret.

### Maternal Commentaries

**FIGURE 2:**
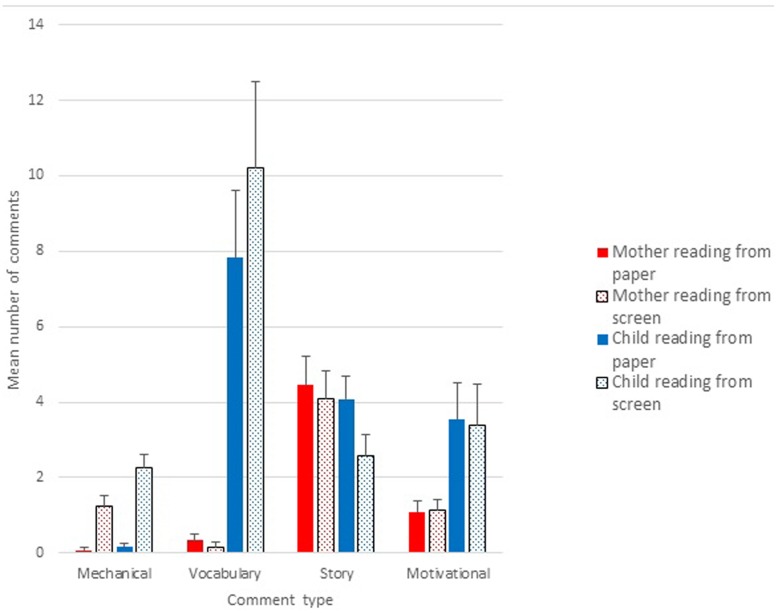
**Proportion of mother comments of each type for reading from paper and screen, with mother or child as reader**.

There were very few ‘unrelated’ comments (fewer than 3% in each condition), so we excluded these from analysis. We computed commentaries as a proportion of the total number of maternal comments, to control for differences in verbosity between conditions, as shown in **Figure 2**, and compared the effects of medium and reader using ANOVA, for each comment type. As we anticipated, mechanical comments were confined almost entirely to reading from screens rather than books, *F*(1,23) = 25.70, *p* < 0.001, with no influence of who was reading and no interaction, *F*s < 1. Again as we would expect, mothers provided more commentary on vocabulary when the child was reading than when she read herself, *F*(1,23) = 76.08, *p* < 0.001. There was no difference between paper and screen for such comments, and no interaction between medium and reader, both *F* < 1.

Commentary about the story was reasonably frequent overall, but differed according to condition: there was a main effect of who was reading, *F*(1,23) = 26.65, *p* < 0.001, with more comments on the story when mother, rather than child, was the reader. Although not significant, we should note that the main effect of medium yielded *F*(1,23) = 3.50, *p* < 0.07, with more story comments for paper than for digital.

There were no significant effects of medium or reader for motivational comments, *F*s < 1.

### Postural Synchrony

**FIGURE 3:**
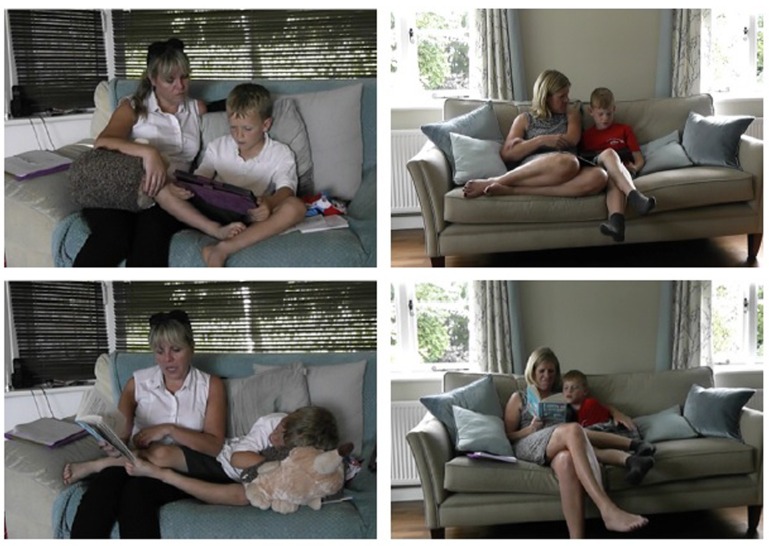
**Curling up with paper, shoulder-surfing with screen: postures for mother reading from paper (bottom) to child reading from screen (top)**. Consent was obtained for use of these images.

All but two sessions involved mother and child sitting side-by-side on a sofa, holding the reading device either jointly or singly. Despite this uniformity, there was a range of ways that pairs divided the work of holding the device, turned pages, shared attention between text and partner, made themselves comfortable, and arranged themselves in relation to the device and their partner. These factors also changed between the different conditions, with partners altering their positions as device or reader shifted. We did not find a single method of coding these different features, since each pair had their own means of altering their differing postural relationships, but inspection of stills of the typical posture in each separate condition for each pair shows that the main contrast was between children reading on screens and mothers reading on paper. When children read from a screen they tended to hold the tablet in a ‘head-down’ posture typical of solo uses such as one-player games or surfing the internet (**Figure 3**, top left and top right). Temporal analysis of the videotapes shows that this ‘head-down’ starting position meant that mothers found it hard to share the screen, leading them to curl round behind the child in order to ‘shoulder-surf’ the screen, rather than adopting the ‘curled up’ position common when reading the paper book (**Figure 3**, bottom right). In contrast, when a mother read from paper, she often held the book between herself and the child, with the child very close to her, either tucked under her arm to facilitate visual sharing (**Figure 3**, bottom right) or in a very relaxed posture with audio sharing but little sight of the book (**Figure 3**, bottom left).

**FIGURE 4:**
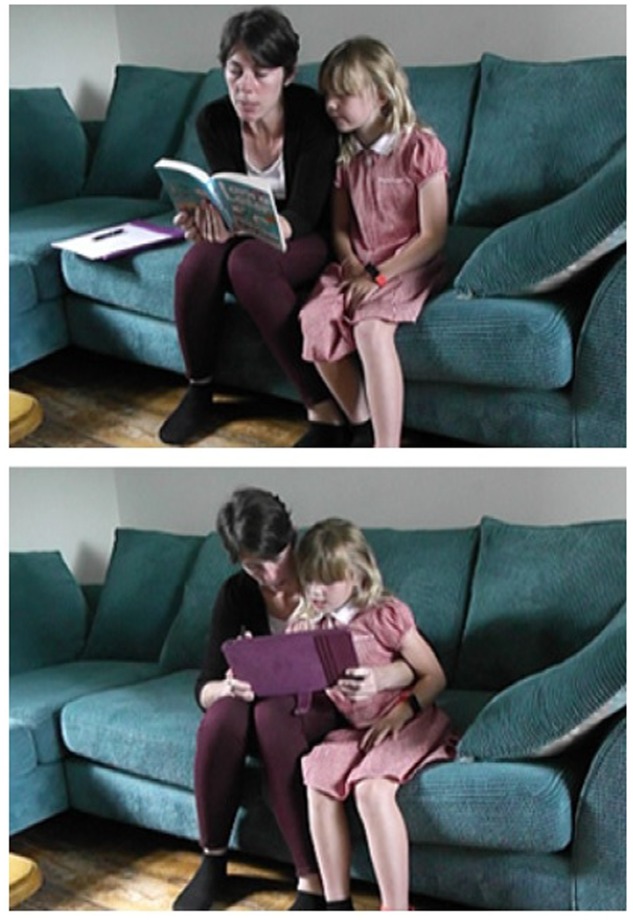
**Atypical pattern of surfing with paper, curling up with screen.** Consent was obtained for use of these images.

Mothers were seen to shift their positions between ‘curled up with paper’ and ‘shoulder-surfing with tablet’ or finding other ways to stretch to see the screen, to accommodate the different ways that children negotiated use of the reading device. We should note that this ‘curling up’ with the paper book, compared to ‘shoulder-surfing’ with a screen, was common but not universal, with one pair atypically closer together when the child was reading from the tablet, and more separate with mother reading the paper book. (**Figure 4**). However, this child was one of the youngest and therefore needed more help when reading.

### Attitudes and Digital Experience

Mothers overwhelmingly expressed a preference for reading on paper, whether this was for reading themselves or for their child as reader, as shown in **Table 2**. Children were more mixed in their opinions, being fairly evenly split regardless of reader. Of the children, seven consistently preferred screen and nine consistently chose paper, and there was a slight tendency for paper to win out when the child was reading, with four children preferring paper for their own reading and screen for their mother reading and only one showing the reverse pattern. There were no marked gender differences in these figures.

**Table 2:** Expressed preferences for paper or screen by reader, for mothers and children. (*N* = 24, with remainder of participants expressing no preference).

	Mother reading		Child reading	
Medium	Paper	Screen	Paper	Screen
Respondent
Mother	22	0	21	2
Child	11	11	13	10

All children (except 1 non-responder) reported having access to a tablet or computer plus television at home, and most had more devices than these. Twenty-two of 24 children used at least 1 available digital device for games, but only one child mentioned use of such a device for reading, despite all children reporting reading every day. Ten of the children reported reading at home with a parent, all using paper, not screens. Sixteen children reported reading mostly fiction, and only one child reported exclusively reading factual books.

For children’s reported activity at school, 16 of 24 reported reading there every day, and all but one of these was exclusively on paper. Tablets (largely iPads) and/or laptops were in reported use at school for 19 of the 24 children, largely for educational games. No child reported shared story reading on a tablet either at school or in the home.

## Discussion

Many studies have investigated differences in children’s experience of shared reading from screens vs. paper, but primarily addressing only individual cognitive factors. Furthermore, the majority focus only on parent-led shared reading, where the child is being read to by the adult, rather than both parent-led and child-led shared reading. We observed both types of shared reading to examine not just cognitive measures of recall and story engagement, but also measures designed to capture other aspects that we believed were important to the child’s experience of shared reading: interaction warmth, parent commentary, postural synchrony, habits and attitudes to technology. We summarize the main findings of our study, and their novelty, and then discuss each of these aspects in turn, followed by remarks about design and questions for further research.

We found that reading interactions involving a screen showed slightly but significantly lower warmth than those with a paper book, and warmth dropped over time for screens, particularly when children rather than mothers took the role of reader. Further, children showed higher story engagement with paper than with screen, and there was suggestive evidence that mothers also made more story-relevant comments with paper books. The two media were associated with different positioning for mother and child: a qualitative analysis suggested that child readers held and used tablets in ways more typical of individual use, so that mothers had to ‘shoulder-surf’ the screen, whereas mothers read paper books in ways that supported shared visual attention, enabling the child to adopt a range of curled-up postures. We found no differences in narrative and descriptive aspects of story recall for stories shared on paper or screen, whether the mother or child was reading.

Child-led shared reading showed different patterns from mother-led reading: children were more engaged with the story when they read themselves rather than when their mothers read, and mothers provided differentiated commentary, with more vocabulary support and less story-focused commentary when the child was reading than when reading herself. Mothers almost exclusively preferred reading from paper, for themselves and for their child, while the results for children’s preferences were more mixed. Despite this, in their everyday practice parents and children reported almost always reading on paper, whether alone or during shared reading.

Our study demonstrates the value of using a broader array of measures based on a wider appreciation of the factors that influence children’s experience of naturalistic shared reading in everyday settings. In the interests of providing a reading experience as typical, smooth, and motivating as possible, we allowed children a choice of books and had them read in each condition with the same book, meaning that we did not control for content or amount read across the sample. However, the identity of the book did not appear to have any systematic influence on the results, and we believe that the choice and freedom of movement provided for participants enabled us to see an illuminating variety of physical synchrony between mother, child and reading device that informed our analysis. However, our sample was small, very homogeneous and in a narrow age-range, so deserves replication and extension with a more diverse range of groups, settings, and texts.

The context of early reading is a shared one during which children gradually develop into independent readers. Our results demonstrate that, in light of this, it is important to consider not just the potential cognitive influences of paper vs. screen (e.g., recall), but also whether the reading medium influences wider cognitive properties such as engagement with the story, and interactional aspects such as warmth. We suggested that the affective differences we found were linked to the different physical positioning of mother and child in paper and screen reading. Our results demonstrate the validity of this approach, given that reliable and significant differences were identified in the extended measures, while we found no difference in standard cognitive aspects of recall.

We now turn to discussing each aspect of the interaction by medium: recall, attentional engagement, interaction warmth, maternal commentary and postural synchrony, and previous experiences with technology and reading. We also comment on differences between mother- and child-led shared reading and discuss possible implications for design.

Previous studies have shown varying results for the cognitive factors of children’s story recall and comprehension when reading from paper or screen: for example, De Jong and Bus (2002) found better learning of content for pre-schoolers being read to by an adult from a paper book than from an e-book, but Takacs et al. (2014) noted that e-books can support word learning and story comprehension just as well as print stories when they use well-designed multi-media extras. Our study used digital texts with no multimedia extras, in order to compare paper to screens more directly in relation to recall, and yielded no difference by reading medium. Mothers provided fairly frequent commentary about the stories in all conditions, and this high level of support might have reduced any differences in recall that might otherwise have occurred. In our study, we used only a 1 to 3 scale of narrative coherence and descriptive richness, to allow comparison of children who had read different amounts of text. It may be that more nuanced measures, and measures across longer time periods, would pick up subtler differences in qualities of recall than recall counts alone. For example, Mangen and Kuiken (2014) found that adults reading text on an iPad self-reported lower narrative coherence than readers on paper. Given the mixed results on recall for screen reading, it seems that any such differences are neither simple nor compellingly large.

Our results on interactive warmth are novel. Although we did not predict the lower warmth for screen reading, it was consistent with the pattern of results from our other measures. Reading on screens was associated with lower engagement of the child with the story and elicited a higher proportion of maternal comments about the mechanics of reading. There was a hint (not significant) of fewer maternal story-related comments for shared screen reading, a pattern also suggested by results of some previous studies. We suggested that the different postural arrangements of mother and child with the different media might support these different qualities of interaction. These findings deserve further research.

Our observations of the reading sessions suggested that posture, and how readers held the reading device, influenced the tenor of the interaction. The typical posture for an adult when children read on screen was a ‘shoulder-surfing’ one, which seems to be a consequence of the fact that when children are actively engaged with reading from the screen, their body position tends to be perched, head down, over the screen in a way that makes it difficult for the adult to see or join in – even in the present study where we used a device in landscape format with similar dimensions to the paper book. From our own observations and experience working with children sharing devices, we have found that children are often reluctant to cede control of a digital device, perhaps because they justifiably see themselves as ‘digital natives’, an impression supported in this study both by mothers’ comments about their children’s use of screens and by children’s commonly expressed preference for reading from screen (see also Yuill et al., 2013 on children sharing iPads). Books seem not to present the same impulse for control: when the pair read a paper book, it seemed natural to open the pages wider to invite the listener to curve inwards and share. When the adult read on paper, we observed that children sometimes adopted a more passive back-seat role, curling up under the mother’s arm or stretching out, sometimes not even in view of the book, but listening, with their upper limbs no longer poised to hold the book or to act, e.g., to turn pages. It may be that these postures more closely reflect their role if shared reading happens at bedtime, with the child lying in bed, distant from the book. Such behaviors will reflect both the cultural practices and habits tied to the reading device – for example, the primarily individual use of tablets – but also the physical properties of the device in relation to its use. Thus, the tablet we used was considerably heavier than the book, and so some children found it easier to hold it in both hands, so a child who needed a hand free to run a finger under the line of text had to manage the device differently. We propose that differences in posture reflect both physical properties of the devices and the powerful cultural practices and habits tied to the devices. The way the device is held has implications for how easy the device is to share, and this can influence the closeness of the interaction.

We now turn to implications for design and further research. Our study is novel in addressing child-led shared reading, a context that is common during children’s extended transition to independent reading. It is notable that children showed more engagement with the story when they were reading themselves than when being read to, although our design did not enable us to see whether this difference was associated with differences in recall or comprehension. It seems plausible that story memory might be better when the adult reads, given the effort required by these emerging readers when required to decode the text themselves. This is a question for future research. As we might expect, mothers gave different verbal support when the child was reading than when she read herself: children were given help with vocabulary and decoding when they read, perhaps leading to a relatively small number of comments about the story content itself. Thus, the identity of the reader taps different requirements, even though, for paper at least, interactions appear equally warm with either reader. Our sample all volunteered for the study, so are likely to be families comfortable with shared reading, and results might be different with other samples, and indeed with other family members, such as fathers.

The number of parents reading to their children seems to reduce sharply during the transition to independent reading, and adult reading is generally a solo activity. Designing e-books for sharing has therefore not been a primary focus. E-books can provide digital traces of previous readers, such as text items highlighted and definitions checked, unlike print books, but e-books do not capitalize on the interactive processes that are typically part and parcel of children’s shared book reading experience when they curl up to read a good book.

Children in our study, in common with many other children, used tablets and laptops very extensively both at home and at school. They also generally read on paper daily, and with enthusiasm, both alone and with their parents, sharing reading roles. However, the use of digital technology and the activity of reading seemed to exist in two somewhat separate spheres. Children were fairly evenly divided between how much they reported enjoying their experiences of reading on screen and on paper during the study. However, this did not reflect their customary reading practices, for which they overwhelmingly reported preferring paper. These self-reported preferences are reflected in our child engagement findings: children were rated as more engaged in shared reading from paper than from screens, and when they were reading rather than being read to. This may suggest that, because digital devices are so often used in solo situations (in contrast to the typically shared use of books in the early years), reading books on digital devices moves from a potentially shared activity to a more individual, private activity.

If digital texts are to be used for shared reading, then their features could be designed to support this more effectively. Krcmar and Cingel (2014) report some frustration experienced by adults using e-books for shared reading, and several studies (e.g., Lauricella et al., 2014), including our own, report more parental talk about the mechanics of reading for screen than for paper. Our e-reader was not designed with the needs of emerging readers in mind. In particular, children’s imperfect control and coordination of eye movements means that they often find it helpful to run their finger below the line of text they are reading: clearly this can prove frustrating with many e-readers, as it will produce unintended effects such as accidental page-turns. Even basic digital features can prove distracting: for example, some children were intrigued by the electronic page-turning effects, with a child in our pilot work becoming particularly engaged with playing with the page turn function to produce interesting shapes on the screen. Page-turning was mentioned by some children as a feature they enjoyed, and by others as a source of frustration. Mothers’ views were less variable, with many reporting that the automatic page turn function hampered their child when decoding unfamiliar words. Thus, features that were designed to remain in the background can become unexpectedly foregrounded. Their visibility can be exacerbated by the fact that there is no single standard for how e-texts operate.

The way readers arranged themselves physically round a reading device may affect how easily an adult can support young readers’ word decoding. For example, an adult sitting side by side with a child can observe the child’s finger traversing the words, see head posture, share the visual field and hear attempts to sound out a word, enabling them to provide help that is sensitive to the child’s particular difficulty. E-readers could perhaps be designed to underpin adult support better, or to provide audio-visual cues to support synchronization of adult help in shared reading.

Children reading to adults is a typical part of early literacy development but has been rarely examined in the context of digital books, given that most research has involved younger and less accomplished readers. Comparison of children sharing paper and digital books at the transitional age of 7 to 9 is of practical import, as children in the immediately foreseeable future will need to gain independent literacy skills, even if they have access to audio-provided e-books for individual reading. Given that shared reading can clearly provide a warm and comfortable context for parent-child interaction, its potential role in fostering collaborative activity and shared emotional experience is worth considering, particularly in a context where digital media could reduce face-to-face sharing. Where everyone has their own device, there is less opportunity for co-watching and co-experiencing, but shared reading, for example, with the traditional bedtime story, provides such an opportunity.

Comparison of digital and print media is not a one-dimensional experimental variable defined by the physical properties of books or touchscreens. Each medium comes with its own set of affordances and cultural practices: for example, the models of how we acquire, archive and share digital vs. print media are quite distinct. We can lend paper books to as many friends as we like, while we may be restricted to a single loan with electronic media; we need to take a trip to the library to borrow a paper book, but can just log in to our account to borrow an e-book; a paper book tends to have a single purpose (being read, maybe being used as a paperweight or door wedge) while an e-book is often only one app on a highly multi-functional device that can also be used to book tickets, play games, work on spreadsheets, and watch films. Further, there are physical differences between books and screens, such as weight, that we can expect to influence the embodied experience of shared reading. The role of such differences is increasingly recognized in embodied approaches to cognition and interaction (Thelen and Smith, 1994).

The cultural significance of devices is a useful reminder that studies of children’s e-reading are being carried out during a time of very rapid technological change: for example, light, flexible screens will change reading postures markedly, altering shared reading in new ways. In earlier studies of e-readers, the technology has tended to be novel, and hence perhaps motivating, and this novelty factor may be less compelling in more recent studies. Our sample, for example, were all very familiar with tablets: indeed, some parents commented on how pleasant novelty of sharing a paper book with their children. In line with previous studies (e.g., Lauricella et al., 2014), we found greater frequency of ‘mechanical’ comments about the process of reading in the screen condition, and this is to be expected when operation of the technology, e.g., of page-turning, is familiar, but less stable than the equivalent mechanism in a paper book. It is important to consider specific design of the technology in studies of digital literacy: for example, mouse interfaces (as in Lauricella et al., 2014) provide very different mechanisms of shared control than touchscreens.

Our findings of differences in warmth over time for paper versus screen reading, and the suggested influence of physical properties and cultural affordances of screens shows the value of considering shared reading and digital text in terms broader than just the cognitive. In particular, differences in warmth are of interest given the powerful role of parent-child relationship quality for a whole range of cognitive and social outcomes (O’Connor and Scott, 2007). Studying shared reading in terms of cognition, affect, posture and embodied interaction, with an eye to the cultural practices of reading devices, should help us understand and design better reading experiences as part of children’s development into independent reading in the context of their family relationships.

## Ethics Statement

This study was carried out in accordance with the recommendations of the University of Sussex Sciences and Technology C-REC with written informed consent for adult and child from all adult participants and assent from their children. Adult participants gave written informed consent in accordance with the Declaration of Helsinki. The protocol was approved by the University of Sussex Sciences and Technology C-REC.

## Author Contributions

NY planned the main research questions and methods, analyzed the data and drafted the paper. AM planned the precise details of method, was responsible for recruitment, data collection, data input, video coding schemes and reliability, and provided material and commentary for drafts.

## Conflict of Interest Statement

NY received financial support from Egmont Publishing for a separate pilot study using 3 texts provided by Egmont, which was carried out otherwise as independent research. The method of the current study was informed by that study and used two of the same texts, but the work reported in the current paper was carried out entirely independently. The other author declares that the research was conducted in the absence of any commercial or financial relationships that could be construed as a potential conflict of interest.
